# Supplementary material for: Synergistically Boosting Li Storage Performance of MnWO4 Nanorods Anode via Carbon Coating and Additives
Source: Materials (Basel). 2024 Sep 24;17(19):4682. doi: 10.3390/ma17194682 (PMC11478062; doi:10.3390/ma17194682)
Supplement: Supplementary file 1 [file materials-17-04682-s001.zip › materials-3114617-supplementary.pdf]

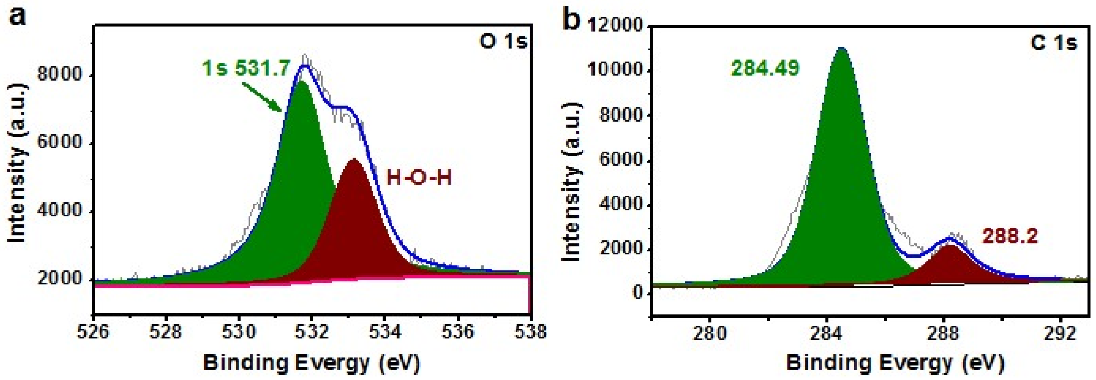


Figure S1. XPS spectra of $MnWO_4$@C, (a) O 1s, (c) C 1s.

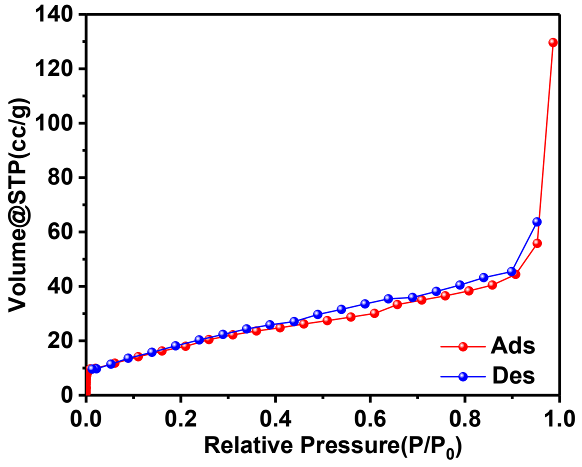


Figure S2. $N_2$ adsorption and desorption isotherms of $MnWO_4$.

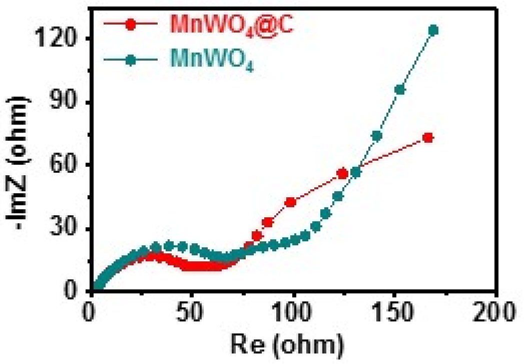


Figure S3. Nyquist plot of MnWO4 and MnWo4@C.

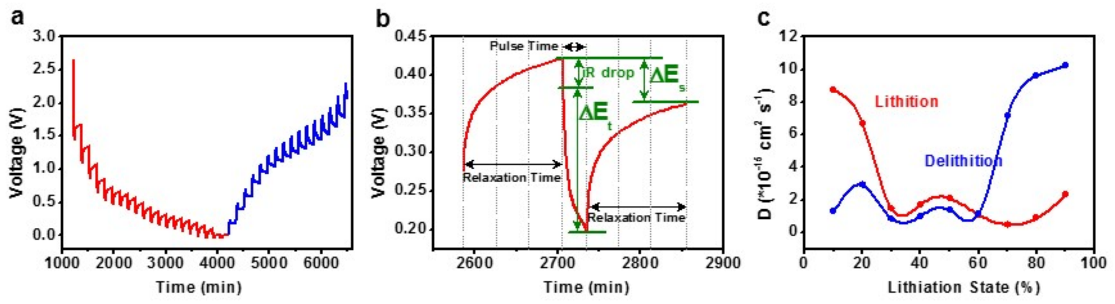


Figure S4. GITT curves of the $MnWO_4$ electrode (discharge/charge state). (b) E vs t profile for one GITT test. (c) DLi+ of MnWO4 during the charge and discharge processes.

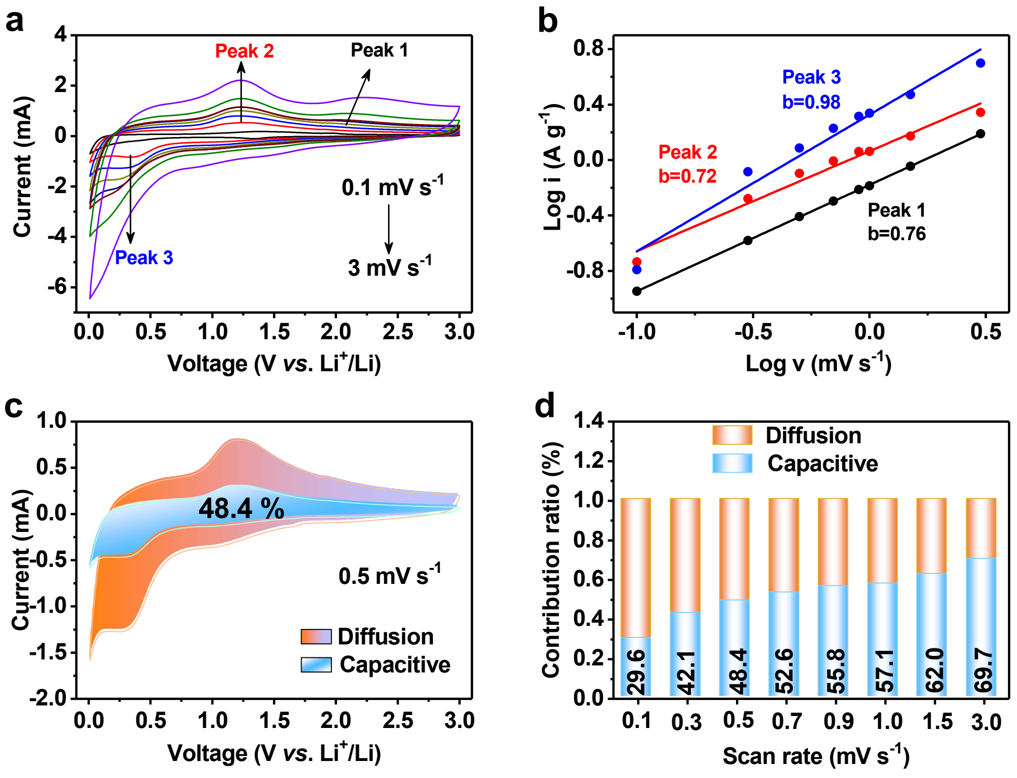


Figure S5. Kinetic analysis of $MnWO_4$ as LIB anodes. (a) CV curves of $MnWO_4$ at different scan rates, (b) log i versus log v plots at each redox peak, contribution ratio of the capacitive and diffusion-controlled charge at (c) 0.5mV $s^{-1}$ and (d) different scan rates.

Table S1.

| | $MnWO_4$ | $MnWO_4$@C |
|---|---|---|
| lithiation states | DLi+ (10-15 $cm^2$ $s^{-1}$) | DLi+ (10-15 $cm^2$ $s^{-1}$) |
| 10 | 8.713 | 21.575 |
| 20 | 6.686 | 1.517 |
| 30 | 1.438 | 7.14 |
| 40 | 1.72 | 5.944 |
| 50 | 2.091 | 3.758 |
| 60 | 1.086 | 4.302 |
| 70 | 0.459 | 2.102 |
| 80 | 0.8637 | 1.563 |
| 90 | 2.327 | 1.758 |

Table S2.

| | $MnWO_4$ | $MnWO_4$@C |
|---|---|---|
| delithiation states | DLi+(10-15 $cm^2$ $s^{-1}$) | DLi+ (10-15 $cm^2$ $s^{-1}$) |
| 10 | 10.25 | 15.42 |
| 20 | 9.592 | 12.1 |
| 30 | 7.167 | 8.46 |
| 40 | 1.167 | 3.377 |
| 50 | 1.408 | 2.747 |
| 60 | 1.013 | 4.254 |
| 70 | 0.8384 | 4.987 |
| 80 | 2.92 | 5.94 |
| 90 | 1.308 | 0.1473 |

**Table S3.** Comparison of electrochemical performance in LIBs with previous works.

| Samples | Rate (C) | Cycle number | Capacity (mAh $g^{-1}$) | $D_{Li^+}$ ($cm^2$ $s^{-1}$) | Refs. |
|---|---|---|---|---|---|
| $MnWO_4$ nanobars (LIBs) | 0.1 | 160 | 600 | | S1 |
| $MnWO_4$ nanoparticles (LIBs) | 0.2 | 150 | 340 | $10^{-19}$-$10^{-20}$ | S2 |
| $MnWO_4$@MWCNTs (LIBs) | 0.2 | 30 | 425 | | S3 |
| $MnWO_4$@C (LIBs) | 0.1 | 100 | 1063 | | S4 |
| F-doped nano-$MnWO_4$ (LIBs) | 0.2 | 150 | 200 | $10^{-19}$-$10^{-20}$ | S5 |
| $MnWO_4$@C (LIBs) | 1 | 200 | 600 | $10^{-15}$ | **This work** |

## References

[S1] En Zhang, Zheng Xing, Ji Wang, Zhicheng Ju, Yitai Qian, Enhanced energy storage and rate performance induced by dense nanocavities inside $MnWO_4$ nanobars, RSC Advances, 2 (2012) 6748-6751.

[S2] Wei Wang, Na Wu, Jin-Ming Zhou, Feng Li, Yu Wei, Tao-Hai Li, Xing-Long Wu, $MnWO_4$ nanoparticles as advanced anodes for lithium-ion batteries: F-doped enhanced lithiation/delithiation reversibility and Li-storage properties, Nanoscale, 10 (2018) 6832-6836.

[S3] Hyun-Woo Shim, Ah-Hyeon Lim, Jae-Chan Kim, Gwang-Hee Lee, Dong-Wan Kim, Hydrothermal realization of a hierarchical, flowerlike $MnWO_4$@MWCNTs nanocomposite with enhanced reversible Li storage as a new anode material, Chem. Asian J, 8 (2013) 2851-2858.

[S4] Ge Gao, Wei Dang, Huimin Wu, Guangxue Zhang, Chuanqi Feng, Synthesis of $MnWO_4$@C as novel anode material for lithium ion battery, Journal of Materials Science: Materials in Electronics, 29 (2018) 12804-12812.

[S5] Jianyu Wei, Jinxiu Ma, Wei Wang, Taohai Li, Na Wu, Dabin Zhang, Study of the effect of F-doping on lithium electrochemical behavior in $MnWO_4$ anode nanomaterials, Journal of Inorganic and Organometallic Polymers and Materials, 31 (2021) 3175-3182.
